# High Prevalence of Long COVID in Common Variable Immunodeficiency: An Italian Multicentric Study

**DOI:** 10.1007/s10875-024-01656-2

**Published:** 2024-02-06

**Authors:** Annalisa Villa, Cinzia Milito, Carla Maria Deiana, Renato Finco Gambier, Alessandra Punziano, Helena Buso, Patrick Bez, Gianluca Lagnese, Giulia Garzi, Giulia Costanzo, Gloria Giannuzzi, Chiara Pagnozzi, Virgil A. S. H. Dalm, Giuseppe Spadaro, Marcello Rattazzi, Francesco Cinetto, Davide Firinu

**Affiliations:** 1 Department of Molecular Medicine, “Sapienza” University of Rome, Rome, Italy; 2 Department of Medical Sciences and Public Health, University of Cagliari, Monserrato, Italy; 3 Rare Diseases Referral Center, Internal Medicine I, Ca’ Foncello Hospital, AULSS2 Marca Trevigiana, Department of Medicine - DIMED, University of Padova, Treviso, Italy; 4 Department of Translational Medical Sciences, University of Naples Federico II, Naples, Italy; 5 Department of Internal Medicine, Division of Allergy & Clinical Immunology, Erasmus University Medical Center, Rotterdam, the Netherlands; 6 Department of Immunology, Erasmus University Medical Center, Rotterdam, the Netherlands

**Keywords:** Long COVID, common variable immunodeficiency, SARS-CoV-2, COVID-19, complicated phenotype, chronic lung disease, obesity

## Abstract

The long-term effects of SARS-CoV-2 infection represent a relevant global health problem. Long COVID (LC) is defined as a complex of signs and symptoms developed during or after SARS-CoV-2 infection and lasting > 12 weeks. In common variable immunodeficiency (CVID) patients, we previously reported higher risk of hospitalization and death during SARS-CoV-2 infection, as well as prolonged swab positivity and frequent reinfections. The aim of the present study was to assess the risk of LC in an Italian cohort of CVID patients. We used a translated version of the survey proposed by Centers for Disease Control and Prevention (CDC) to collect data on LC. In the enrolled cohort of 175 CVID patients, we found a high prevalence of LC (65.7%). The most frequent LC symptoms were fatigue (75.7%), arthralgia/myalgia (48.7%), and dyspnea (41.7%). The majority of patients (60%) experienced prolonged symptoms, for at least 6 months after infection. In a multivariate analysis, the presence of complicated phenotype (OR 2.44, 95% CI 1.88–5.03; *p* = 0.015), obesity (OR 11.17, 95% CI 1.37–90.95; *p* = 0.024), and female sex (OR 2.06, 95% CI 1.09–3.89; *p* = 0.024) significantly correlated with the development of LC. In conclusion, in this multicenter observational cohort study, we demonstrated that CVID patients present an increased prevalence of LC when compared to the general population. Improved awareness on the risk of LC in CVID patients could optimize management of this new and alarming complication of SARS-CoV-2 infection.

**Supplementary Information:**

The online version contains supplementary material available at 10.1007/s10875-024-01656-2.

## Introduction

COVID-19 affected more than 770 million people (772,166,517 people at last update) worldwide since February 2020 and resulted in millions of hospitalizations and more than 6.9 million deaths (6,987,831 deaths at last update), representing a global health emergency (https://www.who.int/emergencies/diseases/novel-coronavirus-2019/situation-reports, last access December 22nd 2023) [1]. The long-term effects of SARS-CoV-2 infection in the COVID-19 disease survivors have been less explored than the acute phase of infection, but it represents a relevant global health problem involving millions of people worldwide with social and economic repercussions [2]. Post-acute sequelae of various infections in the general population have been extensively reported in literature, in particular after Epstein-Barr virus, Giardia lamblia, and other coronavirus infections (SARS-CoV and Middle East respiratory syndrome-CoV) [3–5]. According to the World Health Organization (WHO), long COVID (LC), also known as post-acute sequelae of COVID-19 (PASC), or post COVID-19 condition is defined as the presence of signs and symptoms developed during or after COVID-19 and continuing for more than 12 weeks, not explained by an alternative diagnosis [6].

Since different definitions of LC are available and different study designs were used, it is difficult to estimate the real prevalence of this condition worldwide. According to the WHO, 10 to 20% of adults infected with SARS-CoV-2 eventually develop LC (https://www.who.int/europe/news-room/fact-sheets/item/post-covid-19-condition; last access December 22, 2023) [7]. The most recent data from the Center for Disease Control and Prevention (CDC) on the US population report that LC affects 11% of adults previously infected with SARS-CoV-2 [8]. Epidemiological data on LC in the Italian population are not available. LC symptoms include fatigue, cough, dyspnea, chest pain, palpitation, headache, anosmia, dysgeusia, musculoskeletal symptoms like myalgias and arthralgias, brain fog, and other cognitive disorders. Moreover, neuropsychiatric dysfunctions, such as depression, anxiety, and sleep disturbance, have been described [9], as well as gastrointestinal (diarrhea, nausea), renal (hematuria, oliguria), endocrine alterations (amenorrhea), and dermatological manifestation (cutaneous rash) [10]. LC symptoms generally affect daily activity with an important negative effect on health-related quality of life (HRQoL) [11]. A meta-analysis showed that female sex is a risk factor for the development of LC [12]. In the general population, older age (> 65 years old), obesity and cardiovascular comorbidities, and chronic obstructive pulmonary disease (COPD) and asthma were found to be risk factors for LC [10, 13, 14]. A severe course of SARS-CoV-2 infection and the duration of hospitalization were linked to a higher odds of LC, while vaccination was protective [15–18]. Of note, patients with LC show an impaired formation of anti-SARS-CoV-2 neutralizing antibodies and anti-Spike protein and lower levels of IgG3 antibodies [19, 20].

Inborn errors of immunity (IEIs) are a group of heterogeneous disorders resulting in increased susceptibility to infections, development of autoimmune conditions, and increased risk of malignancy [21]. Higher hospitalization and mortality due to COVID-19 have been reported in this group of patients when compared to the general population in different studies [22–24].

Within IEIs, common variable immunodeficiency (CVID) is the most frequent symptomatic disorder in adults [25]. Despite the higher susceptibility to infections, studies focusing on their post-acute sequelae in CVID are lacking. Besides a higher risk of hospitalization and death during SARS-CoV-2 infection, these patients experienced prolonged course of COVID-19 and a higher rate of reinfection [26, 27].

Thus, these patients could be prone to develop LC, taking into account a potential impaired antibody response and a high prevalence of pre-existing lung damage. However, to date, no study addresses LC in patients with primary antibody deficiencies. We performed a retrospective observational cohort study with the aim to determine the prevalence of LC in an Italian multicentric cohort of CVID patients. Secondly, we tried to define general and specific risk factors for the development of LC.

## Methods

We performed a multicentric retrospective observational cohort study including a cohort of consecutive patients with a CVID diagnosis established according to the European Society for Immunodeficiencies (ESID) criteria [28]. The population was selected from four Italian referral centers for primary immune deficiencies of Rome, Naples, Padua, and Cagliari. The study period lasted from March 1, 2020, to June 1, 2023. The inclusion criteria were diagnosis of CVID, age ≥ 18 years, a documented SARS-CoV-2 infection, and a follow-up ≥ 6 months after infection. SARS-CoV-2 positivity was assessed by approved tests (PCR molecular test and rapid antigen test with determination of cut off index). Genotype assessment of SARS-CoV-2 variant on nasopharyngeal swabs was not systematically conducted. The infection strain was attributed mostly on the basis of the date of the positive test, according to the epidemiological data available for National Health Authorities of Italy [29, 30]. The duration of SARS-CoV-2 infection was recorded as the days from the first positive swab to the first negative one. Reinfection was defined as the record of a new positive SARS-CoV-2 test > 90 days after the resolution of the first infection. Data about sex, age, body mass index (BMI), CVID clinical phenotype, comorbidities, ongoing therapies, COVID-19 disease severity, vaccination status, and SARS-CoV-2-specific treatments, such as antiviral therapy or monoclonal antibodies (mAbs), were collected. Considering BMI values, the population was divided into underweight (BMI < 18.5), normal weight (BMI from 18.5 to 24.9), overweight (BMI from 25.0 to 29.9), and obese (BMI ≥ 30.0). BMI presented is the habitual value, preceding SARS-CoV-2; data was assessed also after infection, and no variations were found in our cohort. Comorbidities recorded included obesity, arterial hypertension, previous cardiovascular events, chronic arterial disease, diabetes mellitus, chronic kidney disease (CKD), and malignancy. The SARS-CoV-2 infection’s complications reported were hospitalization, pulmonary thromboembolism (PTE), and bacterial superinfections. Chronic lung disease was considered in the presence of any pre-existing chronic pulmonary involvement, in particular bronchiectasis and/or granulomatous lymphocytic interstitial lung disease (GLILD) and/or end-stage lung disease (ESLD) and/or asthma and/or COPD. ESLD was defined as chronic oxygen-dependent respiratory failure. Chronic immunosuppressive treatment was considered in the presence of chronic administration of steroids and/or disease-modifying antirheumatic drugs (DMARD) and/or mAbs for immunosuppressive purposes. The CVID cohort was classified into two different clinical phenotypes according to Chapel’s criteria: “infection only” and “complicated’’ [31]. All patients enrolled in the study were on IgG replacement therapy (IgRT), and IgG trough level (IgG TL) was included in the laboratory data collection. COVID-19 severity was defined according to WHO classification [32]. Due to the small sample size, moderate and severe COVID-19 disease were merged in a single group (moderate-severe patients), including also intensive care unit (ICU) admission. Data about thoracic computed tomography were not available for the entire cohort, so not included in the study. All vaccinated patients received a mRNA vaccine, namely, BNT162b2. The dates of the first positive and the first negative SARS-CoV-2 test were recorded to evaluate the duration of the RT-PCR positivity.

According to WHO definition, LC was defined as “Signs and symptoms that develop during or after SARS-CoV-2 infection and continue for more than 12 weeks and are not explained by an alternative diagnosis” [33]. The survey proposed by CDC was used to collect data on LC [34]. Figure S1 shows the Italian translated version of CDC long COVID survey. The symptoms evaluated in the survey were fatigue, mental fog, difficulty breathing/shortness of breath, joint/muscle pain, palpitations, chest pain, dizziness, changes in the menstrual cycle (for female patients), difficulty in tasting and smelling, and inability to exercise. After obtaining informed consent, the questionnaire was offered to patients by trained health professionals, during an outpatient or a telematic visit. The LC questionnaire data collection took place from October 1, 2022, to June 1, 2023. The study was approved by the Local Ethical Committee and was performed in accordance with the most recent version of the Declaration of Helsinki.

### Statistical Analysis

Patient characteristics were summarized using medians, standard deviations, interquartile ranges (IQR), and percentages as appropriate. Chi-squared tests of independence or Fisher’s exact tests were used for categorical data as appropriate. Mann–Whitney *U* was used for unpaired continuous data. Binomial logistic regression models were fitted to calculate odds ratios (OR) with 95% confidence intervals (95% CI). To confirm the finding, multivariable logistic regression analysis was performed considering age, sex, SARS-CoV-2 infection course, and considerable CVID features. Statistical significance was considered as a *p* value < 0.05. All the analyses were performed using Jamovi version 2.3.28.0.

## Results

A total of 224 CVID patients who experienced SARS-CoV-2 infection were enrolled in this study from March 1, 2020, to June 1, 2023. Since eight patients died during SARS-CoV-2 infection or in the next few months and two patients were lost at follow-up, the CDC LC survey was offered to 215 patients with 175 responders (81% of the total cohort). The median interval of time of follow-up since SARS-CoV-2 infection to the date of completion of the CDC survey was of 13 (IQR 11–16) months. The sex and age distribution of responders and general cohort were similar. Of note, 115/175 patients (65.7%) presented at least one symptom of LC (LC cohort). Patients without evidence of LC symptoms were included in the non-LC cohort.

### Long COVID Features

In the LC cohort, 74 patients (64.3%) were females with a median age of 51 years (IQR 44.0–59.5). CVID clinical phenotype was complicated in 58 patients (50.4%) whereas 53 patients (46.1%) suffered from chronic lung disease and 44 (38.3%) showed bronchiectasis, 18 (15.7%) GLILD, and 2 (1.7%) met the criteria for ESLD. In addition, 35 patients (30.4%) presented, in their history, at least one episode of autoimmune cytopenia, in particular 32 (27.8%) immune thrombocytopenia (ITP) and five patients (4.3%) autoimmune hemolytic anemia (AIHA), two patients suffering of both conditions. CVID-related enteropathy was present in 16 patients (14.3%) of our LC cohort, while systemic autoimmune disease in nine patients (8.0%). Data about demographic features of the study population are summarized in Table 1. In the LC cohort, 83 patients (72.2%) got infected during the Omicron period and the median duration of infection was 18 days (IQR 11.0–23.5). At first infection, 31 patients (27%) were unvaccinated, while 59 patients (51.3%) were vaccinated with 3 doses and 17 (14.8%) with 4 doses. The COVID-19 severity was mild in 104 patients (90.4%), while 12 (9.6%) presented a moderate-severe infection that required hospitalization, and two patients (1.7%) required ICU admission. The complications reported during SARS-CoV-2 infection were PTE in one patient (0.9%) and bacterial superinfections in six patients (5.2%). Data about SARS-CoV-2 course of infection are summarized in Table 2.

**Table 1:** Demographics, CVID-related features, and comorbidities of LC and non-LC cohort

Survey responders (*n* = 175) (*n* (%))	LC (115 (65.7))	Non-LC (60 (34.2))	*p*	OR (95% CI)
Demographics				
Median age (IQR)	51 (44–59.5)	48.5 (33–62.8)	0.273	1.01 (0.99–1.03)
Sex (F) (%)	74 (64.3)	28 (46.7)	**0.024**	**2.06 (1.09–3.89)**
Median BMI (IQR)	23.5 (21.2–27)	23.6(21–25.7)	0.137	1.09 (1.01–1.19)
BMI underweight (%)	5 (4.4)	10 (17.2)	**0.008**	**0.22 (0.07–0.68)**
BMI normal range (%)	65 (57.0)	28 (48.3)	0.207	1.50 (0.80–2.84)
BMI overweight (%)	30 (26.3)	19 (32.8)	0.514	0.79 (0.40–1.59)
BMI obesity (%)	13 (11.4)	1 (1.7)	**0.028**	**7.34 (0.93–57.5)**
CVID clinical phenotype				
Complicated phenotype (%)	58 (50.4)	18 (30)	**0.010**	**2.37 (1.22–4.60)**
Autoimmune cytopenia (%)	35 (30.4)	7 (11.7)	**0.002**	**3.94 (1.55–10.00)**
ITP (%)	32 (27.8)	6 (10)	**0.007**	**3.47 (1.36–8.85)**
AIHA (%)	5 (4.3)	3 (5)	0.845	0.86 (0.20–3.74)
Enteropathy (%)	16 (14.3)	15 (25.4)	0.072	0.49 (0.22–1.08)
Immunosuppressive therapy (%)	12 (10.4)	3 (5)	0.233	2.21 (0.60–8.17)
Laboratory features				
Median lymphocyte count (IQR)	1470 (1170–2060)	1545 (1167–2205)	0.939	1.00 (0.99–1.00)
Patients with IgA < 7 (%)	56 (41.1)	26 (44.8)	0.594	0.84 (0.45–1.60)
IgG TL (IQR)	708 (554–854)	719 (543–859)	0.939	1.00 (0.99–1.00)
Respiratory involvement				
Chronic lung disease (%)	53 (46.1)	21 (35.0)	0.160	1.59 (0.83–3.03)
GLILD (%)	18 (15.7)	6 (10)	0.302	1.67 (0.63–4.46)
Bronchiectasis (%)	44 (38.3)	18 (30)	0.278	1.45 (0.74–2.82)
ESLD (%)	2 (1.7)	0 (0)	0.547	2.67 (0.26–56.4)
Comorbidities				
Obesity (%)	13 (11.4)	1 (1.7)	**0.028**	**7.34 (0.93–57.5)**
Hypertension (%)	28 (24.3)	13 (21.7)	0.691	1.16 (0.55–2.46)
Diabetes mellitus (%)	10 (8.7)	3 (5)	0.376	1.81 (0.48–6.84)
Cardiovascular events (%)	6 (5.2)	3 (5)	0.951	1.05 (0.25–4.34)
Arterial disease (%)	9 (7.8)	5 (8.3)	0.907	0.93 (0.30–2.92)
CKD (%)	3 (2.6)	3 (5)	0.409	0.51 (0.10–2.60)
Cancers (%)	16 (13.9)	12 (20)	0.297	0.65 (0.28–1.47)

Statistically significant results in bold

**Table 2:** SARS-CoV-2 course of infection of LC and non-LC cohorts

Survey responders (*n* = 175)	LC (*n* = 115)	Non-LC (*n* = 60)	*p*	OR (95% CI)
Unvaccinated (%)	31 (27)	12 (21.7)	0.303	1.50 (0.69–3.27)
Vaccination status 3 doses (%)	59 (51.3)	31 (51.7)	0.325	0.70 (0.35–1.42)
Vaccination status 4 doses (%)	17 (14.8)	12 (20)	0.380	0.69 (0.31–1.57)
Median days of infection (IQR)	18 (11–23.5)	13 (8–22)	**0.029**	**1.02 (0.99–1.05)**
Wuhan + alpha	25 (21.7)	8 (13.3)	0.181	1.81 (0.76–4.29)
Delta	7 (6.1)	3 (5)	0.769	1.23 (0.31–4.94)
Omicron (%)	83 (72.2)	50 (83.3)	0.104	0.52 (0.23–1.15)
Antiviral (%)	37 (32.2)	17 (28.3)	0.602	1.20 (0.60–2.38)
mAb (%)	46 (40)	19 (31.7)	0.280	1.44 (0.74–2.78)
Antiviral + mAb (%)	78 (67.8)	36 (60.0)	0.303	1.41 (0.73–2.69)
Mild (%)	104 (90.4)	59 (98.3)	0.050	0.16 (0.02–1.27)
Moderate-severe (%)	12 (9.6)	1 (1.7)	0.050	6.24 (0.79–49.51)
Reinfections (%)	26 (22.6)	11 (18.3)	0.510	1.30 (0.59–2.86)
ICU (%)	2 (1.7)	1 (1.6)	0.972	1.04 (0.93–11.8)
Complication during COVID-19				
Hospitalization (%)	12 (9.6)	1 (1.7)	0.050	6.24 (0.79–49.51)
PTE (%)	1 (0.9)	0	1.000	1.59 (0.64–39.5)
Bacterial superinfection (%)	6 (5.2)	1 (1.7)	0.255	3.25 (0.38–27.6)

Statistically significant results in bold

### Comparison of Long COVID Cohort Versus Non-long COVID Cohort

In our study, we found significant associations of LC with female sex (OR 2.06, 95% CI 1.09–3.89; *p* = 0.024), clinical complicated phenotype (OR 2.37, 95% CI 1.22–4.60; *p* = 0.010), and clinical history of autoimmune cytopenia (OR 3.94, 95% CI 1.55–10.00; *p* = 0.002). Data are shown in Table 1. Obesity (BMI ≥ 30.0) was significantly more represented in the LC cohort, but with a wide OR and 95% CI (OR 7.34, 95% CI 0.93–57.5; *p* = 0.028). On the other hand, hospitalization tends to be related to the development of LC (OR 6.24, 95% CI 0.79–49.5; *p* = 0.050), whereas mild disease seems to be a protective factor (OR 0.16, 95% CI 0.02–1.27; *p* = 0.050), albeit none of the associations reached statistical significance. Complete data are presented in Table 2.

**Fig. 1:**
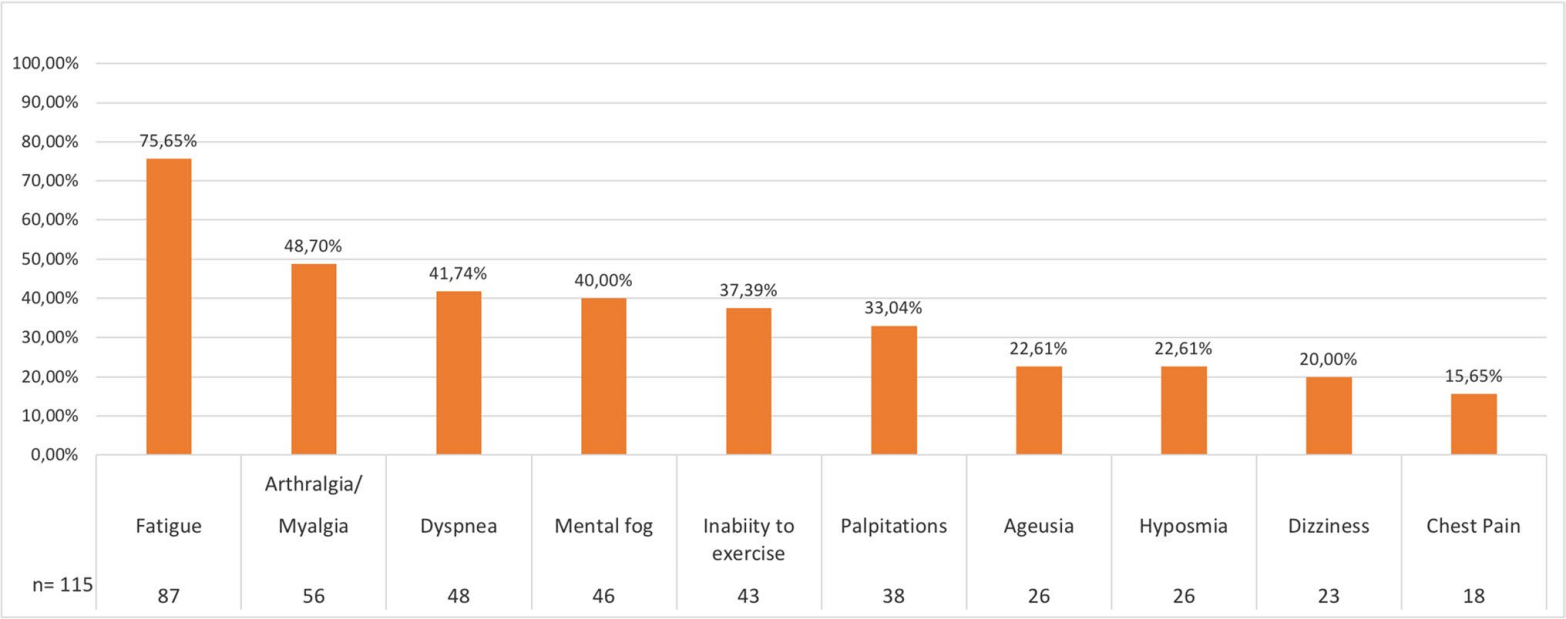
Long COVID symptoms with number and percentage of patients considering the LC-CVID cohort in our population (*n* = 115)

The most frequent symptoms reported in the LC survey were fatigue in 87 patients (75.7%), arthralgia/myalgia in 56 patients (48.7%), and dyspnea in 48 patients (41.7%). Of note, 89 patients (77.39%) had at least three symptoms and 66 patients (57.4%) had more than three symptoms of LC. Interestingly, the LC symptoms lasted more than 6 months in 69 patients (60%) and 59 patients (51.3%) declared persistence of symptoms at the moment of the present survey. Symptoms of LC in our cohort are summarized in Fig. 1. We then explored the possible correlation of single LC symptoms and LC duration with CVID-related features and SARS-CoV-2 course of infection. We found significant results regarding fatigue, dyspnea, and symptoms lasting more than 6 months. Complete data are resumed in Supplementary Table S1. Fatigue was significantly related to complicated phenotype (OR 2.35, 95% CI 1.10–5.02; *p* = 0.027), autoimmune cytopenia (OR 2.74, 95% CI 1.31–5.74; *p* = 0.007), and ITP (OR 2.70, 95% CI 1.26–5.79; *p* = 0.009). Interestingly, vaccination with four doses had a protective effect on the development of fatigue (OR 0.39, 95% CI 0.17–0.92; *p* = 0.028). Not surprisingly, dyspnea correlated with chronic lung disease (OR 2.39, 95% CI 1.21–4.71; *p* = 0.011), GLILD (OR 3.92, 95% CI 1.61–9.51; *p* = 0.002), and bronchiectasis (OR 2.34, 95% CI 1.18–4.63; *p* = 0.013). Moreover, the presence of dyspnea correlated with female sex (OR 2.41, 95% CI 1.17–4.98; *p* = 0.016), complicated phenotype (OR 2.60, 95% CI 1.31–5.14; *p* = 0.005), and a moderate-severe infection course (OR 6.15, 95% CI 1.76–21.51; *p* = 0.004). Prolonged symptoms, lasting more than 6 months, were related to female sex (OR 2.45, 95% CI 1.29–4.67; *p* = 0.006) and moderate-severe course of infection (OR 5.02, 95% CI 1.31–19.25; *p* = 0.019). We then repeated the analysis considering only the Omicron wave. Results are summarized in Supplementary Table S2. Of note, neither vaccination status nor SARS-CoV-2-specific treatments impacted on the LC development in the Omicron period. On the other hand, autoimmune cytopenia persisted to significantly correlate to LC development (OR 4.15, 95% CI 1.34–12.87; *p* = 0.014). Univariate analysis for LC adjusted for sex and age was performed in order to better define significant data. Significant variable were female sex (OR 2.00, 95% CI 1.05–3.80; *p* = 0.034), obesity (OR 8.83, 95% CI 1.02–70.80; *p* = 0.040), BMI underweight (OR 0.17, 95% CI 0.05–0.56; *p* = 0.003), and complicated phenotype (OR 2.28, 95% CI 1.17–4.46; *p* = 0.016), autoimmune cytopenia (OR 3.67, 95% CI 1.32–10.16; *p* = 0.012), and in particular ITP (OR 3.28, 95% CI 1.27–8.44; *p* = 0.014). Infection during the Omicron period was instead associated with a lower risk of LC development (OR 0.40, 95% CI 0.17–0.92; *p* = 0.032). Interestingly, in a multivariate analysis considering sex, age, complicated phenotype, hospitalization, chronic lung disease, days of duration of the SARS-CoV-2 infection, obesity (BMI ≥ 30.0), and Omicron period, only complicated phenotype (OR 2.44, 95% CI 1.88–5.03; *p* = 0.015), obesity (OR 11.17, 95% CI 1.37–90.95; *p* = 0.024), and female sex (OR 2.46, 95% CI 1.20–5.07; *p* = 0.014) significantly correlated with the development of LC. For completed data, see Table 3.

**Table 3:** Multivariate analysis: CVID variables/SARS-CoV-2 infection features and LC

Survey responders (*n* = 175)	*p*	OR (95% CI)
Sex (F)	**0.014**	**2.46 (1.96–5.07)**
Complicated phenotype	**0.015**	**2.45 (1.20–5.03)**
Obesity (BMI > 30.0)	**0.024**	**11.17 (1.37–9.95)**
Age	0.418	1.01 (0.98–1.04)
Hospitalized	0.211	4.10 (0.45–36.71)
Omicron	0.191	0.53 (0.21–1.37)
Median days of infection	0.930	0.99 (0.96–1.03)
Chronic lung disease	0.715	1.41 (0.56–2.31)

Statistically significant results in bold

## Discussion

In our CVID cohort, we found a high prevalence of LC, particularly in those patients with a complicated phenotype. LC is emerging as a frequent complication of SARS-CoV-2 infection; however, research on the topic is still limited. LC has an important impact on HRQoL of patients who survived the acute phase of the infection. Multiple chronic diseases, such as arterial hypertension, diabetes, obesity, COPD, and asthma, lead to an increased risk of severe COVID-19 and may contribute to the development of LC during the phase of recovery [10, 13, 14]. Indeed, a severe course of SARS-CoV-2 infection with need of hospitalization has been reported to increase the risk of LC [15–17]. In patients with IEIs, there is an increased risk of severe and fatal COVID-19 disease when compared to the general population [22–24]. More specifically, CVID patients have specific risk factors that expose them to a severe and prolonged course of SARS-CoV-2 infection and a high rate of reinfection [26, 27]. Chronic lung disease, a complicated clinical phenotype, and chronic immunosuppressive treatment were found to be associated with an increased risk of COVID-19-related hospitalization and death [22, 26, 27]. The CVID cohort analyzed in the present study reflects the epidemiology of CVID cohorts described in literature, presenting a younger median age than the general population and an increased incidence of respiratory involvement, autoimmune complications, and malignancy [35]. Also, the higher number of patients infected during the Omicron wave reflects the spreading of the infection in the general population, likely related to higher transmission capacity of the strain and to the reduction in vigilance after vaccination and reduction of disease severity.

To our knowledge, this is the first multicentric retrospective observational cohort study to assess the prevalence and the specific features of LC in patients with CVID. In our CVID cohort, prevalence of LC seems to be higher than in the general population (65.7% of the cohort who responded to the survey vs 11% reported in the general adult US population or 10–20% reported by the WHO) (https://www.who.int/europe/news-room/fact-sheets/item/post-covid-19-condition; last access December 22, 2023) [7, 8].

Pathogenesis of LC is mostly unknown, and data about pathophysiological mechanisms are limited. Impaired vaccine response, with defective neutralizing antibody production, has been implied in the development of LC in the general population [19]. A reduced formation of neutralizing antibodies can be present in CVID patients, and we could speculate that this incomplete response may, at least in part, explain the increased prevalence of LC in our CVID patients compared to the general population, even if serological data were not available in our cohort [36].

In our CVID cohort, females were found to have an increased risk of developing LC (64.3% of the LC-cohort). This result is in line with available data in the general population [12]. Less severe acute COVID-19 disease in females has been linked to estrogen functions, downregulation of ACE-2 expression, and higher expression of type I IFN [37]. However, an altered regulation of type I IFN may lead to overactivation of the immune system, predisposing females to higher risk of LC symptoms after recovery of infection [38].

Regarding SARS-CoV-2 infection features, a severe course requiring hospitalization leads to an increased probability of prolonged symptoms and LC, especially in vulnerable individuals [16, 17]. In our cohort, hospitalization during infection tends to have an impact on LC development, even if not reaching statistical significance. In a previous study, we demonstrated that complicated phenotype is a risk factor for hospitalization in CVID patients during SARS-CoV-2 infection [26]. In the present study, LC correlates with complicated phenotype in CVID patients. Apart from obesity, different from the general population, cardiovascular comorbidities and chronic lung disease seem not to impact LC development in our CVID cohort [16]. Of note, Hajjar et al. in 2017 suggested that higher BMI is generally related to the presence of fatigue in primary antibody deficiency (PAD) patients [39]. On the other hand, in our cohort, BMI underweight was significantly more prevalent in the non-LC group. To our knowledge, data about the relation between underweight and LC are not present in literature, while nutritional deficiencies have been related to risk of hospitalization, death, and LC sequelae in patients with severe COVID-19 [40]. Underweight is a multifactorial condition that might be influenced by nutritional status and enteropathy in CVID patients. However, we did not find any difference in enteropathy between LC and non-LC subgroups, and data about nutritional status were not available. Further studies including a multidimensional evaluation of nutritional status could be useful to better characterize the relation to LC development.

Due to the younger age of CVID patients, cardiovascular events are not frequent complications in our cohort, probably explaining the absence of correlation. Moreover, in our CVID cohort, chronic lung disease did not influence LC prevalence. Conversely, the item of dyspnea in the LC survey, defined as new-onset dyspnea after SARS-CoV-2 infection, was significantly related to a pre-existing chronic lung involvement.

In the multivariate analysis, considering age, chronic lung disease, SARS-CoV-2 infection features such as severity, duration of infection, and Omicron period, only female sex, obesity, and complicated phenotype were significantly linked to LC in our CVID cohort. Although pathogenesis of LC is still mostly unclear, immunological dysfunction with persistence of chronic inflammation seems to be involved [5]. The persistence of high levels of IFN I and IFN III and proinflammatory cytokines, such as CXCL9, CXCL10, and IL-8, after COVID-19 recovery has been related to the development of LC [38]. Interestingly, a similar cytokine dysregulation has been described in CVID patients with a complicated phenotype and autoimmune cytopenia [41].

The impact of autoimmune disease on LC development is controversial. Despite the known role of COVID-19 as a trigger of chronic inflammation and immune dysregulation in predisposed individuals, the role of pre-existing immune dysregulation on LC risk is debated [42]. Inflammatory rheumatic diseases have been associated with LC development in different cohorts, suggesting that chronic inflammation of systemic immune-rheumatic diseases impact on post-acute sequelae of COVID-19 [43]. However, patients with inflammatory rheumatic diseases experience chronic symptoms such as myalgia, arthralgia, and fatigue that in part overlap with LC. For this reason, the observed difference in these patients when compared with healthy controls could in part be explained by clinical manifestations in the context of underlying rheumatic diseases [44].

Post-acute sequelae of other infectious diseases have been explored in non-CVID patients, in which chronic complications of infections often occur as persistent organ specific symptoms such as inflammatory bowel disease-like presentation after Giardia lamblia infection [4] or as chronic fatigue syndrome after EBV or Middle East respiratory syndrome (MERS) infection [45]. Symptoms of LC could in part overlap with chronic fatigue syndrome; however, post-acute sequelae of COVID-19 can be accompanied by rheumatological symptoms, dizziness, loss of smell and taste, palpitations, and due to the multisystem nature of this condition. CVID patients are predisposed to several infections with the risk of recurrence and chronic evolution. However, apart from EBV-related lymphomas, studies focusing on post-acute sequelae of infections in CVID are lacking [46, 47].

Dyspnea, fatigue, and arthralgia/myalgia were the most frequent LC reported symptoms in our CVID cohort, and 57.4% of our patients had more than three LC symptoms. Dyspnea and fatigue are known complications that worsen the quality of life of patients with chronic conditions and primary immunodeficiencies [39, 48]. Discrimination of long COVID symptoms in the context of chronic diseases and vulnerable individuals may be challenging. In our cohort, dyspnea was not surprisingly correlated with the presence of a chronic lung disease. However, the CDC survey specifically explored new-onset symptoms after COVID-19. In our cohort, we did not register lung function worsening but just an increase of the reported subjective symptom “dyspnea.” We can speculate that SARS-CoV-2 perturbed the respiratory balance, especially in fragile individuals with pre-existent pulmonary diseases. Moving to fatigue, apart from autoimmunity, in our cohort, this symptom did not correlate with female sex, obesity, and bronchiectasis; as previously reported to be strongly correlated with this specific symptom in CVID patients before the pandemic [39], this might support a more LC-related than CVID-related explanation of fatigue in our patients. Finally, despite dyspnea and fatigue could be potentially influenced by pre-existing comorbidities, arthralgia and myalgia are not CVID-specific features.

Unfortunately, a specific pre-COVID evaluation of all the symptoms possibly confounding with LC was not available in the described CVID cohort. However, data collected before the pandemic in our and in other CVID cohorts report a prevalence of tiredness, weakness, arthralgia lower than 25%, and a prevalence of fatigue around 30%, which is much less than what found in our LC cohort [39, 49]*.* This further supports the LC-related etiology of the symptoms reported in our study.

Due to the heterogeneity of symptoms, lack of defined pathophysiological mechanisms, and non-standardized diagnostic criteria, a specific treatment for LC is not defined. However, prompt individuation of LC risk factor in vulnerable patients could optimize follow-up and future management, also considering physical therapy, psychological support, and rehabilitation [50, 51].

We are aware that this study has some limitations. First of all, the relatively small sample size and the not standardized LC diagnosis could have an impact on the selection of the LC cohort in the study. Second, the use of patient-reported outcomes and the possible overlap of LC symptoms with CVID-related symptoms could represent a bias, as for other chronic diseases. We also did not systematically evaluate serological response to SARS-CoV-2 infection and vaccination, and cytokine profile of our CVID patients was not available. Moreover, the retrospective design of the study could also have generated a temporal bias, because of the lapse of time from the SARS-CoV-2 infection and the administration of LC survey. However, the majority of patients experienced prolonged symptoms lasting more than 6 months from the infection and often still present when answering the survey. Moreover, our CVID patients are familiar with CVID-QoL and other QoL-related questionnaires investigating symptoms such as weakness/tiredness, dyspnea, and myalgia. For this reason, when asked through the CDC tool to specifically focus on new-onset symptoms, we believe that they have been able to discriminate them from those previously present in daily life and that the LC-related patient reported outcome is reasonably trustable. To our knowledge, this is the first multicentric observational cohort study trying to assess the role of CVID features on the risk of developing LC. We administered a CDC survey to measure LC symptoms, and our CVID cohort reflects the epidemiology of infection and disease-related complications reported in literature for such a rare disease. Our findings are partly in line with those coming from the healthy population and fragile patients [15, 17].

In conclusion, we demonstrated that CVID patients present an increased prevalence of LC than the general population. In CVID, factors associated with LC are female sex and obesity, like in the healthy controls. In addition, the complicated phenotype, a specific feature of the CVID immune-dysregulated substrate, is associated with LC. An improvement in awareness on the risk of LC condition in CVID patients could optimize the management of a new and alarming complication of SARS-CoV-2 in fragile patients, even if further studies are needed to confirm the findings.

## Supplementary Information

Below is the link to the electronic supplementary material.

Supplementary file1 (DOCX 833 KB)

## Data Availability

Data is available upon reasonable request to the corresponding author.

## Abbreviations

IEI: Inborn errors of immunity
CVID: Common variable immunodeficiency
LC: Long COVID
PASC: Post-acute sequelae of COVID-19
WHO: World Health Organization
CDC: Centers for Disease Control and Prevention
ESLD: End-stage lung disease
GLILD: Granulomatous lymphocytic interstitial lung disease
ITP: Immune thrombocytopenia
AIHA: Autoimmune hemolytic anemia
COPD: Chronic obstructive pulmonary disease
BMI: Body mass index
mAbs: Monoclonal antibodies
ICU: Intensive care unit
HRQoL: Health-related quality of life
IgG-TL: IgG trough level
CKD: Chronic kidney disease
