# Supplementary material for: High Prevalence of Long COVID in Common Variable Immunodeficiency: An Italian Multicentric Study
Source: J Clin Immunol. 2024 Feb 6;44(2):59. doi: 10.1007/s10875-024-01656-2 (PMC10847195; doi:10.1007/s10875-024-01656-2)

**SUPPLEMENTARY DATA**

**Supplementary Tables Legends:**

**Table S1:** Common symptoms of LC in relation to CVID variables, comorbidities and SARS-CoV-2 infection features in the study population.

**Table S2:** LC prevalence in relation to CVID variables, comorbidities and SARS-CoV-2 infection features in the study population during the Omicron period.

**Table S1**

| **LC symptoms** | **fatigue** | | **dyspnea** | | **symptoms>6 months** | |
| --- | --- | --- | --- | --- | --- | --- |
|  | **p** | **OR**  **(95%  IC)** | **p** | **OR**  **(95%  IC)** | **p** | **OR**  **(95%  IC)** |
| **demographics features** |  |  |  |  |  |  |
| **sex F** | 0.054 | 1.82 (0.99-3.34) | **0.016** | **2.41 (1.17-4.98)** | **0.006** | **2.45 (1.29-4.67)** |
| **CVID features** |  |  |  |  |  |  |
| **chronic lung disease** | 0.180 | 0.55 (0.23-1.31) | **0.027** | **2.35 (1.10-5.02)** | 0.939 | 1.03 (0.49-2.18) |
| **GLILD** | 0.639 | 1.23 (0.52-2.92) | **0.002** | **3.92 (1.61-9.51)** | 0.489 | 1.36 (0.58-3.23) |
| **bronchiectasis** | 0.710 | 1.12 (0.61-2.09) | **0.013** | **2.34 (1.18-4.63)** | 0.250 | 1.45 (0.78-2.72) |
| **ESLD** | 0.153 | 5.18 (0.25-109) | **0.021** | **13.7 (0.65-291)** | 0.078 | 7.89 (0.38-167) |
| **complicated phenotype** | **0.028** | **1.97 (1.07-3.61)** | **0.005** | **2.60 (1.31-5.14)** | 0.116 | 1.63 (0.88-3.01) |
| **immunosuppressive treatment** | 0.056 | 3.04 (0.929-9.95) | 0.254 | 1.87 (0.629-5.58) | 0.088 | 2.50 (0.85-7.37) |
| **patients with IgA< 7** | 0.760 | 1.10 (0.60-2.00) | 0.704 | 0.88 (0.45-1.71) | 0.263 | 0.70 (0.38-1.30) |
| **autoimmune cytopenia** | **0.007** | **2.74 (1.31-5.74)** | 0.057 | 2.04 (0.97-4.29) | 0.077 | 1.88 (0.93-3.81) |
| **ITP** | **0.009** | **2.70 (1.26-5.79)** | 0.060 | 2.06 (0.96-4.39) | 0.060 | 2.00 (0.97-4.13) |
| **AIHA** | 0.987 | 1.01 (0.25-4.18) | 0.875 | 0.88 (0.20-4.50) | 0.111 | 0.21 (0.02-1.73) |
| **enteropathy** | 0.176 | 0.58 (0.26-1.28) | 0.817 | 0.90 (0.37-2.18) | 0.989 | 1.01 (0.45-2.24) |
| **comorbidities** |  |  |  |  |  |  |
| **obesity (BMI>30.0)** | 0.217 | 1.89 (0.61-5.90) | 0.954 | 1.04 (0.31-3.48) | 0.791 | 1.16 (0.38-3.51) |
| **hypertension** | 0.395 | 0.74 (0.36-1.49) | 0.618 | 0.81 (0.36-1.82) | 0.761 | 1.12 (0.55-2.28) |
| **diabetes mellitus** | 0.757 | 1.20 (0.38-3.71) | 0.354 | 1.73 (0.53-5.58) | 0.941 | 0.96 (0.30-3.06) |
| **cardiovascular events** | 0.745 | 0.80 (0.20-3.08) | 0.684 | 1.34 (0.32-5.60) | 0.278 | 0.08-2.09) |
| **arterial disease** | 0.562 | 1.38 (0.46-4.17) | 0.920 | 1.06 (0.32-3.57) | 0.767 | 0.84 (0.27-2.63) |
| **CKD** | 0.989 | 1.01 (0.20-5.16) | 0.548 | 0.52 (0.05-4.56) | 0.246 | 0.30 (0.03-2.60) |
| **cancers** | 0.704 | 0.85 (0.38-1.92) | 0.089 | 0.39 (0.12-1.19) | 0.088 | 0.46 (0.18-1.14) |
| **active cancers** | 0.989 | 1.01 (0.20-5.16) | 0.125 | 0.19 (0.01-3.49) | 0.246 | 0.30 (0.03-2.60) |
| **infection features** |  |  |  |  |  |  |
| **unvaccinated** | 0.447 | 1.32 (0.65-2.67) | 0.222 | 1.60 (0.75-3.41) | 0.651 | 1.18 (0.58-2.41) |
| **vaccination status 3 doses** | 0.170 | 0.63 (0.33-1.22) | 0.520 | 0.79 (0.34-1.61) | 0.237 | 0.67 (0.35-1.30) |
| **vaccination status 4 doses** | **0.028** | **0.39 (0.17-0.92)** | 0.072 | 0.37 (0.12-1.13) | 0.153 | 0.53 (0.22-1.28) |
| **omicron** | 0.070 | 0.52 (0.26-1.06) | 0.557 | 0.80 (0.38-1.70) | 0.108 | 0.57 (0.28-1.14) |
| **antiviral** | 0.960 | 1.02 (0.53-1.93) | 0.945 | 1.03 (0.50-2.10) | 0.812 | 1.08 (0.56-2.08) |
| **mAb** | 0.598 | 1.18 (0.64-2.18) | 0.144 | 1.65 (0.84-3.25) | 0.841 | 0.94 (0.50-1.76) |
| **LC symptoms** | **fatigue** | | **dyspnea** | | **symptoms>6 months** | |
| **antiviral+mAb** | 0.461 | 1.26 (0.68-2.36) | 0.331 | 1.43 (0.69-2.93) | 0.846 | 0.94 (0.49-1.77) |
| **exitus** | NA | NA | NA | NA | NA | NA |
| **mild** | 0.224 | 0.47 (0.14-1.62) | **0.002** | **0.16 (0.04-0.57)** | **0.009** | **0.19 (0.05-0.75)** |
| **moderate-severe** | 0.233 | 2.13 (0.62-7.34) | **0.004** | **6.15 (1.76-21.51)** | **0.019** | **5.02 (1.31-19.25)** |
| **reinfection** | 0.182 | 1.65 (0.79-3.44) | 0.951 | 0.97 (0.43-2.20 | 0.361 | 1.41 (0.68-2.92) |
| **complication during covid-19** |  |  |  |  |  |  |
| **hospitalization** | 0.233 | 2.13 (0.62-7.34) | **0.004** | **6.15 (1.76-21.51)** | **0.019** | **5.02 (1.31-19.25)** |
| **PTE** | 0.313 | 3.07 (0.12-76.4) | 0.103 | 8.05 (0.32-201) | 0.214 | 4.66 (0.19-116) |
| **bacterial superinfection** | 0.241 | 2.61 (0.45-13.9) | 0.072 | 3.76 (0.81-17.5) | 0.328 | 2.11 (0.46-9.75) |
| **acute cardiovascular events** | NA | NA | NA | NA | NA | NA |

**Table S2**

| **Survey responders in omicron (n=136)** | **LC** | **non-LC** | **p** | **OR  (95% CI )** |
| --- | --- | --- | --- | --- |
| **n (%)** | 83 (61.0) | 50 (36.7) |  |  |
| **Demographics** |  |  |  |  |
| **median age (IQR)** | 53 (43.5-61.5) | 50 (25-84) | 0.359 | 1.01 (1.99-1.03) |
| **sex F (%)** | 58 (69.9) | 27 (54.0) | 0.066 | 1.98 (0.95-4.09) |
| **median BMI (IQR)** | 23.2 (21-27) | 23.5 (16-29) | 0.262 | 1.09 (0.99-1.20) |
| **CVID clinical phenotype** |  |  |  |  |
| **complicated phenotype (%)** | 39 (47.0) | 15 (30.0) | 0.055 | 2.07 (0.98-4.35) |
| **autoimmune cytopenia (%)** | **22 (26.5)** | **4 (8.0)** | **0.014** | **4.15 (1.34-12.87)** |
| **ITP (%)** | **22 (26.5)** | **5 (10.0)** | **0.027** | **3.25 (1.14-9.23)** |
| **AIHA (%)** | 4 (4.8) | 3 (6.0) | 0.768 | 0.79 (0.17-3.70) |
| **enteropathy (%)** | **10 (12.2)** | **13 (26.5)** | **0.041** | **0.38 (0.15-0.96)** |
| **immunosuppressive therapy (%)** | 9 (10.8) | 2 (4.0) | 0.182 | 2.92 (0.60-14.10) |
| **Laboratory features** |  |  |  |  |
| **median lymphocyte count (IQR)** | 1465 (1178-201) | 1585 (554-850) | 0.407 | 1.00 (0.99-1.00) |
| **patients with IgA< 7 (%)** | 38 (46.3) | 22 (45.8) | 0.955 | 0.98 (0.48-2.00) |
| **IgG TL (IQR)** | 655 (530-850) | 719 (103-1170) | 0.503 | 1.00 (0.99-1.00) |
| **Respiratory involvement** |  |  |  |  |
| **chronic lung disease (%)** | 29 (34.9) | 16 (32.0) | 0.729 | 1.14 (0.54-2.41) |
| **GLILD (%)** | 12 (14.5) | 3 (6.0) | 0.148 | 2.65 (0.71-9.90) |
| **bronchiectasis (%)** | 29 (34.9) | 12 (24.0) | 0.188 | 1.70 (0.77-3.75) |
| **ESLD (%)** | 0 | 0 | NA | NA |
| **Comorbidities** |  |  |  |  |
| **obesity (%)** | 13 (15.7) | 3 (6.0) | 0.110 | 2.91 (0.79-10.77) |
| **hypertension (%)** | 20 (24.1) | 10 (20.0) | 0.585 | 1.27 (0.54-2.99) |
| **diabetes mellitus (%)** | 7 (8.4) | 3 (6.0) | 0.608 | 1.44 (0.36-5.85) |
| **cardiovascular events (%)** | 4 (4.8) | 2 (4.0) | 0.826 | 1.22 (0.21-6.89) |
| **arterial disease (%)** | 7 (8.4) | 3 (6.0) | 0.608 | 1.44 (0.36-5.85) |
| **CKD (%)** | 2 (2.4) | 3 (6.0) | 0.308 | 0.39-2.40) |
| **cancers (%)** | 13 (15.7) | 10 (20.0) | 0.523 | 0.74 (0.30-1.85) |
| **active cancers (%)** | 4 (4.8) | 1 (2.0) | 0.422 | 2.48 (0.27-22.85) |
| **infection features** |  |  |  |  |
| **unvaccinated (%)** | 3 (3.6) | 4 (8.0) | 0.285 | 0.43 (0.092-2.01) |
| **vaccination status 3 doses (%)** | 76 (91.6) | 45 (90.0) | 0.760 | 1.21 (0.36-4.03) |
| **vaccination status 4 doses (%)** | 17 (20.5) | 12 (24.0) | 0.634 | 0.82 (0.32-1.89) |
| **median duration of infection (IQR)** | 15 (10-23.5) | 12.5 (8.0-17.8) | 0.055 | 1.03 (0.99-1.07) |
| **antiviral (%)** | 31 (37.3) | 17 (34.0) | 0.697 | 1.16 (0.55-2.41) |
| **mAb (%)** | 35 (42.2) | 16 (32.0) | 0.244 | 1.55 (0.74-3.24) |
| **antiviral+mAb (%)** | 64 (77.1) | 33 (66.0) | 0.163 | 1.74 (0.80-3.78) |
| **mild (%)** | 81 (97.6) | 50 (100.0) | NA | NA |
| **moderate-severe (%)** | 2 (2.4) | 0 (0.0) | NA | NA |
| **reinfection (%)** | 11 (13.3) | 9 (18.0) | 0.460 | 0.70 (0.27-1.82) |
| **complication during covid-19** |  |  |  |  |
| **hospitalization (%)** | 2 (2.4) | 0 | NA | NA |
| **PTE (%)** | 0 | 0 | NA | NA |
| **bacterial superinfection (%)** | 3 (3.6) | 1 (2.0) | 0.603 | 1.84 (0.19-18.16) |

**Figure S1**

**Supplementary Figure Legend**

**Figure S1:** English and Italian translated version of CDC Long COVID survey


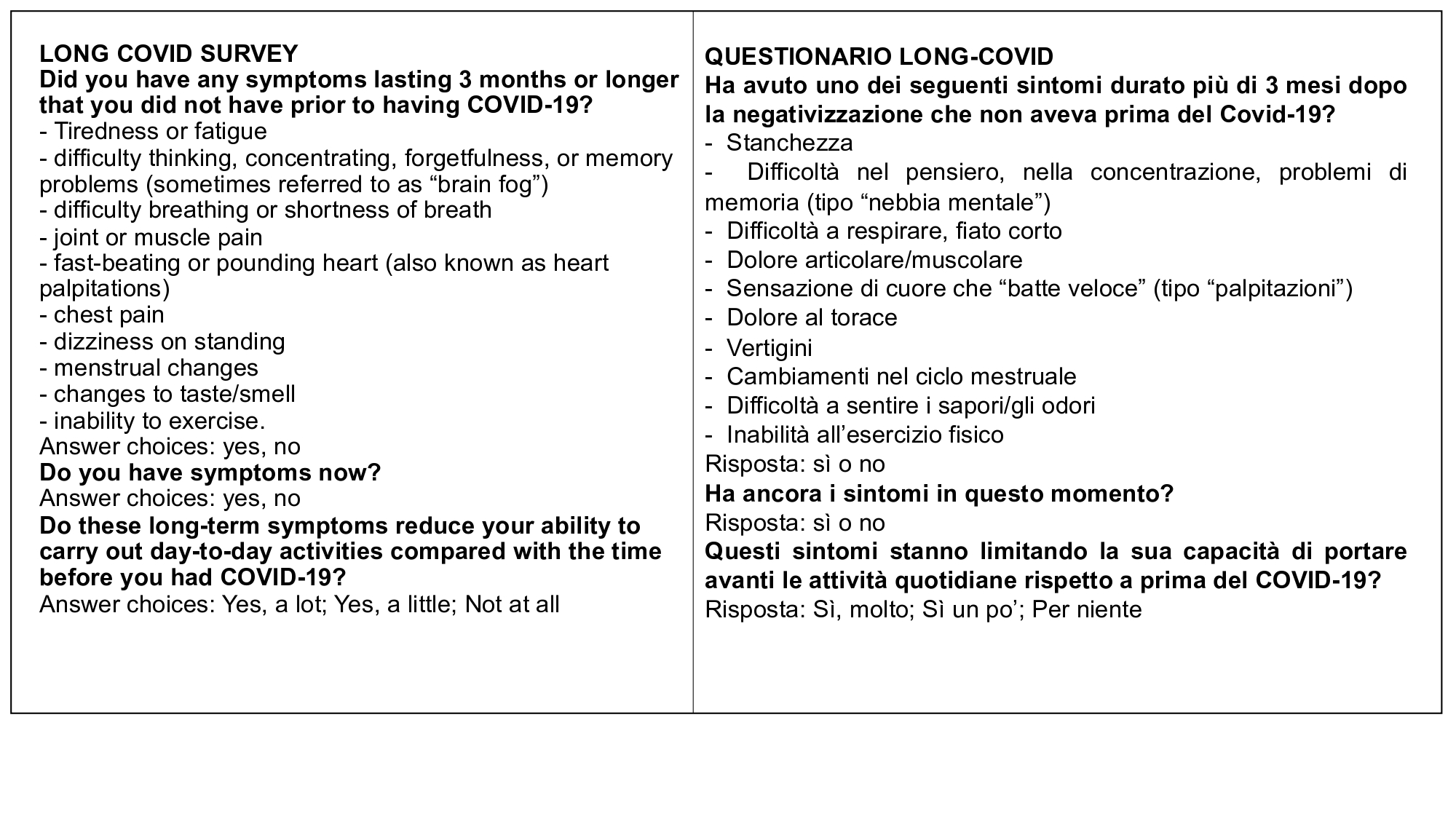

Supplement: Supplementary file 1 — Supplementary file1 (DOCX 833 KB) [file 10875_2024_1656_MOESM1_ESM.docx]
